# Contrasting *Phaseolus* Crop Water Use Patterns and Stomatal Dynamics in Response to Terminal Drought

**DOI:** 10.3389/fpls.2022.894657

**Published:** 2022-05-31

**Authors:** Jose A. Polania, Violeta Salazar-Chavarría, Ingrid Gonzalez-Lemes, Alexis Acosta-Maspons, Caspar C. C. Chater, Alejandra A. Covarrubias

**Affiliations:** ^1^Departamento de Biología Molecular de Plantas, Instituto de Biotecnología, Universidad Nacional Autónoma de México, Cuernavaca, Mexico; ^2^Department of Molecular Biology and Biotechnology, University of Sheffield, Sheffield, United Kingdom; ^3^Royal Botanic Gardens, Kew, Richmond, United Kingdom

**Keywords:** water use efficiency, transpiration efficiency, FTSW threshold, stomatal density, *Phaseolus acutifolius*, *Phaseolus vulgaris*

## Abstract

Terminal drought stress affects more than half of the areas planted with common bean (*Phaseolus vulgaris*), the main food legume globally, generating severe yield losses. Phenotyping water deficit responses and water use are central strategies to develop improved terminal drought resilience. The exploration and exploitation of genetic diversity in breeding programs are gaining importance, with a particular interest in related species with great adaptation to biotic and abiotic factors. This is the case with tepary beans (*Phaseolus acutifolius*), a bean that evolved and was domesticated in arid conditions and is considered well adapted to drought and heat stress. Under greenhouse conditions, using one genotype of tepary beans (resistant to drought) and two of common beans (one resistant and one susceptible to terminal drought), we evaluated phenotypic differences in traits such as water use efficiency (WUE), transpiration efficiency, rate of photosynthesis, photosynthetic efficiency, stomatal density, stomatal index, stomatal size, and the threshold for transpiration decline under well-watered and terminal drought conditions. Our results indicate two different water use strategies in drought-resistant genotypes: one observed in common bean aimed at conserving soil water by closing stomata early, inhibiting stomatal development, and limiting growth; and the other observed in tepary bean, where prolonged stomatal opening and higher carbon fixation, combined with no changes in stomata distribution, lead to higher biomass accumulation. Strategies that contribute to drought adaptation combined with other traits, such as greater mobilization of photoassimilates to the formation of reproductive structures, confer bean drought resistance and are useful targets in breeding programs.

## Introduction

Beans (*Phaseolus* spp.), originating in Mesoamerica and now globally distributed, are of great economic, dietary, and cultural importance (Gepts and Debouck, 1991; Delgado-Salinas et al., 2006; Bitocchi et al., 2017). Among the approximately 70 species in this genus, five are domesticated, of which common bean (*Phaseolus vulgaris* L.) is the most important food legume worldwide, especially in the tropics, where it is the main source of protein, carbohydrates, and minerals, mainly iron and zinc (Beebe et al., 2013, 2014; Rao, 2014). Another domesticated species of this genus is tepary bean (*Phaseolus acutifolius* A. Gray), native to arid areas of northern Mexico and southern United States, which presents resistance to drought, and heat stress, and has been suggested as a model plant for drought resistance in beans (Nabhan, 1985; Mejía-Jiménez et al., 1994; Mohamed et al., 2005; Rao et al., 2013; Bitocchi et al., 2017; Polania et al., 2017; Buitrago-Bitar et al., 2021; Burbano-Erazo et al., 2021).

Beans are generally cultivated by smallholder farmers in the tropics with minimal inputs, and are regularly exposed to unfavorable environmental conditions such as drought (Acosta Gallegos and Kohashi Shibata, 1989; Beebe et al., 2008, 2014). Among the abiotic factors that limit bean production, drought is the most destructive, affecting around 60% of the areas planted with this crop and causing major losses, from 10 to 100% of production, depending on the intensity of stress (Polania et al., 2016a,b; Rao et al., 2017). In Mexican agriculture, beans are almost entirely rain-fed crops and the absence of seasonal rains often results in terminal drought stress, occurring in the reproductive phase of the crop and seriously limiting pod and seed production (Acosta-Gallegos and White, 1995; Arredondo et al., 2020).

The development of bean cultivars resistant to drought through genetic breeding is one of the strategies to tackle this limitation (Beebe et al., 2008; Polania et al., 2016a). In plant breeding, the identification of outstanding bean lines under drought, and the use of drought resistance morpho-physiological traits as selection criteria, play important roles in the efficiency of breeding programs (Reynolds and Trethowan, 2007; Mir et al., 2012). In common beans, various phenotypic characteristics have been reported as linked to drought resistance, such as deep roots, fine roots, osmotic adjustment, stomatal opening control, water use efficiency, effective use of water, and greater mobilization of photoassimilates to pod and seed formation (Cuellar-Ortiz et al., 2008; Rosales et al., 2012; Polania et al., 2017, 2020; Rao et al., 2017; Chater et al., 2019; Polania and Rao, 2019). In tepary bean, drought resistance has been associated to fine roots, water use efficiency, small leaves, osmotic adjustment, sink strength, and greater mobilization of photoassimilates to pod and seed formation (Mohamed et al., 2005; Rao et al., 2013; Polania et al., 2017; Leal-Delgado et al., 2019). The traits reported are dependent on the agroecological conditions, drought type, species, and bean cultivars, among other factors. Nevertheless, a more efficient mobilization of photoassimilates from leaves to pods and seeds, and stomatal control of water use and loss are two distinct processes consistently identified across different ideotypes and bean crop species as associated to drought resistance (Chater et al., 2017, 2019; Polania et al., 2020).

As in most plants, drought leads to reduced photosynthetic rates in beans, thereby slowing or hindering the accumulation of biomass and plant growth (Farooq et al., 2016; Mathobo et al., 2017; Nadeem et al., 2019). To our knowledge there is no evidence of bean genotypes with higher photosynthetic rates under drought, but significant differences in transpiration rate and water use efficiency have been reported (Lizana et al., 2006; Rosales et al., 2012; Polania et al., 2020). Water use efficiency (WUE), a concept introduced a century ago relating plant production to water use, is a useful parameter especially applicable in the sustainable intensification of agriculture that aims for increasingly efficient use of resources, particularly of water (Bertolino et al., 2019; Hatfield and Dold, 2019). WUE is considered an important component in the breeding of drought resistance in different crops; however, it is a complex phenotype governed by many genes and plant processes (Araus et al., 2002; Blum, 2005, 2009; Easlon et al., 2014; Polania et al., 2016a).

Different methodologies for determining WUE have been reported, including measurements from leaf to whole plant levels, as well as from crop canopy to field scale. Each of these methods have advantages and disadvantages, showing sometimes low correlations between leaf and plant level measurements (Medrano et al., 2015; Hatfield and Dold, 2019). Two WUE determinations at leaf level include instantaneous WUE (WUE_i_), defined as the relationship between photosynthetic rate and transpiration rate, and intrinsic WUE (_i_WUE), considered as the relationship between photosynthetic rate and stomatal conductance (Hatfield and Dold, 2019). At plant level, some methodologies require the determination of biomass or production and the water consumed by the plant; an example of this is the transpiration efficiency method (Devi et al., 2009; Vadez et al., 2014). Because leaf temperature also affects transpiration rate, stomatal opening, and therefore water use (Lopes and Reynolds, 2010; Pinto and Reynolds, 2015; Rao et al., 2017), and consequently WUE (Cornic and Ghashghaie, 1991; Lin et al., 2012; Dusenge et al., 2019), there are other WUE determination techniques at plant level that consider ^13^C discrimination and plant infrared thermography (Merlot et al., 2002; Easlon et al., 2014; Polania et al., 2016a).

High WUE has been reported for terminal drought resistant bean genotypes from arid regions under field conditions by using carbon isotope discrimination and canopy temperature depression, as well as stomatal conductance and grain yield (Muñoz-Perea et al., 2007; Beebe et al., 2014; Polania et al., 2016a,b). Studies under controlled conditions, using techniques such as infrared gas analysis to determine WUE_i_, have also shown superior WUE in common bean genotypes resistant to terminal drought (Rosales et al., 2012). Tepary beans, previously shown to be resistant to terminal drought under both field and controlled conditions, have high WUE, and higher WUE than common bean genotypes bred for terminal drought resistance under field conditions (Mohamed et al., 2005; Rao et al., 2013; Polania et al., 2016a).

Foliar and stomatal anatomy, morphology, and physiology also affect WUE parameters. Stomatal density and size affect water use, gas exchange, and therefore photosynthetic rate. Across different species, it has been shown that stomatal density alters WUE. These studies suggest that lower stomatal density (fewer pores per leaf area) combined with smaller stomata allow the plant to save water whilst maintaining gas exchange, resulting in a high WUE under stress conditions (Chater et al., 2016, 2017, 2019; Bertolino et al., 2019).

Stomatal physiology and stomatal opening and closing dynamics also affect WUE, and phenotypic differences in these processes have been reported in many crops, including legumes such as chickpea (*Cicer arietinum*), cowpea (*Vigna unguiculata*), and peanut (*Arachis hypogaea*; Devi et al., 2009; Ratnakumar et al., 2009; Zaman-Allah et al., 2011; Belko et al., 2012). As water becomes less available in the soil, the plant minimizes transpirational water loss by reducing stomatal aperture until stomatal closure is achieved. For most crops, this point (the threshold for a decline in transpiration) has been reported when the fraction of water available for transpiration in the soil is between 0.4 and 0.3 (Sinclair and Ludlow, 1986; Devi et al., 2009; Ratnakumar et al., 2009; Belko et al., 2012; Sinclair, 2012; Vadez et al., 2014). For legumes such as peanut and chickpea, some genotypes gradually close their stomata when they detect soil water depletion, allowing them to save this water in the soil profile; while other genotypes can sustain stomatal apertures for longer under drought, thereby maintaining higher rates of gas exchange and carbon fixation (Devi et al., 2009; Belko et al., 2012).

The phenotypic characterization of stomatal traits of different bean genotypes, as well as the better understanding of molecular and genetic controls, are necessary to interpret the mechanisms involved in the WUE processes, information that will help to subsequently develop elite germplasm resistant to drought stress. Even though WUE has been determined in some common and tepary bean varieties under optimal and drought conditions, few detailed comparative physiological studies have been reported considering WUE and stomatal dynamics in these species. Within this framework, the different sensitivity to terminal drought, between different common varieties, and between tepary beans, the present study aims to phenotypically characterize terminal drought response mechanisms related to WUE, transpiration (including stomatal density and physiology) and photosynthesis between common bean genotypes and between an elite tepary bean and common bean cultivars.

## Materials and Methods

### Plant Materials and Growing Conditions

For this comparative phenotypic analysis, we selected two elite common bean (*Phaseolus vulgaris* L.) genotypes widely cultivated in Mexico: “Bayo Madero” (BM; drought-susceptible) and “Pinto Saltillo” (PS; drought-resistant); both genotypes belong to the Durango race within the Mesoamerican gene pool (Rosales et al., 2012). One tepary bean (*Phaseolus acutifolius* A. Gray) elite drought-tolerant genotype was included, “Tep32” (Porch et al., 2013). The selection of these genotypes was based on our interest to go deeper into those traits that could be associated to drought resistance in the Durango race gene pool, a good source of drought resistance in improvement programs, and into the differences and likeness with tepary bean. We are aware that this is a restricted number of genotypes, nevertheless, this allowed us a more detailed analysis. Plants were grown under greenhouse conditions with an average temperature of 24°C, a relative humidity of 60%, and a natural light/dark cycle at the Instituto de Biotecnología (IBt) of the Universidad Nacional Autónoma de México (UNAM; Cuernavaca, Morelos, Mexico). Greenhouse- and pot-based experiments were undertaken, following previous experiences in our lab (Cuellar-Ortiz et al., 2008; Rosales et al., 2012; Polania et al., 2020). This approach ensures replicability and repeatability of experiments by controlling substrate composition for dry-down, removing the effects of Mexico’s seasonal rains, and avoiding confounding variables, such as herbivory and pathogens. Two terminal drought experiments were conducted: one designed to determine shoot biomass accumulation and photosynthetic responses, and another to determine differences in transpiration efficiency and decline of transpiration. The methodological details of each experiment will be described below. In both experiments, seeds were sown in pots (21 cm × 16 cm × 15.5 cm, 5 L) containing a mix of vermiculite:perlite:Metromix (4:3:3). Osmocote fertilizer was applied at planting and flowering time.

### Shoot Biomass Accumulation and Photosynthesis Traits Experiment

A randomized complete design with two watering treatments (well-watered and water deficit to simulate terminal drought), one plant per pot and six biological replicates were used. Plants were grown under optimal irrigation during vegetative development. At flowering time (approximately 30 days after planting, phenology of the three genotypes under greenhouse conditions was closely aligned), plants were subjected to the two watering treatments: well-watered (WW) where pots were irrigated up to field capacity, and water deficit treatment where pots were maintained at 25% of field capacity to simulate terminal drought (TD). TD was imposed from flowering to harvest (mid-pod-filling stage). Irrigation, in both well-watered and water deficit conditions, was carried out daily.

#### Shoot Biomass Accumulation

To determine differences in the accumulation of vegetative and reproductive biomass between the evaluated genotypes, sampling was carried out at the mid pod filling stage (60–70 days after planting), considering the phenological stage when the plant reached maximum plant vigor (Polania et al., 2016a). To avoid loss of biomass due to plant natural defoliation, sample collection was performed when plants entered the stage of physiological maturity. Plants were cut at ground level, and samples were stored in paper bags and immediately dried in an oven at 60°C until sample weights remained constant. Dry shoot biomass (stem, leaves, and pods) was expressed in grams (g) per plant. The percentage of shoot biomass reduction due to water deficit treatments was calculated using biomass data from well-watered and terminal drought samples.

#### Gas Exchange Measurements and Photosynthetic Parameters

Gas-exchange measurements were conducted on one fully expanded leaf for six plants in each treatment between 10 am and 12 pm using an infrared gas analyzer (LI-6400; LI-COR) at the mid-pod-filling stage. Photosynthesis was induced with saturating light (1,000 mmol photons m^2^ s^−1^) and 400 μmol mol^−1^ CO_2_ in the leaf chamber. These conditions were kept constant for the determination of net photosynthetic rate (AN), transpiration rate (E), stomatal conductance (gs), and instantaneous water-use efficiency (WUE_i_) measurements. WUE_i_ was calculated as the ratio between the photosynthetic rate and transpiration rate. At the same stage of development, leaf relative chlorophyll content (SPAD units) and the photosynthetic efficiency of photosystem II in light-adapted leaves (Fv/Fm ratio) were determined in a fully expanded leaf using a MultispeQ (PhotosynQ; Kuhlgert et al., 2016) in six plants per treatment.

### Stomata Density and Stomata Size Experiment

To determine phenotypic differences in stomatal traits including stomatal density, index, and size, an additional experiment was established using identical watering treatments to those described above; one plant per pot and five biological replicates were used. Plants were grown under optimal irrigation during vegetative development. At flowering time (30 days after planting), plants were subjected to the two watering treatments: well-watered (WW), where the pots were irrigated up to field capacity, and water deficit, where pots were maintained at 25% of field capacity from flowering to mid-pod-filling stage to simulate terminal drought (TD). Irrigation in both control and water deficit conditions was carried out daily.

To interrogate the effects of growth stage on bean leaf water deficit responses, the plant canopy was considered and analyzed as three sections: the basal section (B), which contains the oldest and most mature leaves; the middle section (M), in which there is a mixture of mature and growing leaves; and the apical section (A), where most leaves are actively growing (Supplementary Figure S3). To determine stomata density, stomata index, and size, two expanded leaflets from independent trifoliates per section (basal, middle, and apical) per plant were selected for each treatment and replication. Epidermal impressions were obtained by using dental impression media (Imprint II Garant light body by 3 M ESPE) on the underside from leave central part, covering about 2 cm^2^ with the mixture, letting it set and then removing. Subsequently, the negative impressions were covered with clear nail varnish, subsequently peeled off using Crystal Clear Scotch tape and affixed to a glass microscope slide for microscopic observation. Stomatal size (length and width) and stomatal density (later converted to stomata per mm^2^) were counted across 20 fields of view, the average value was obtained from the measurements in the 20 fields per section for each genotype and treatment. Stomatal index (SI) was calculated by dividing the number of stomata per field by the total number of cells per field (stomata + other epidermal cells) and multiplying by 100. Stomatal length and width are reported independently. Stomatal width was obtained by measuring guard cells in their middle region, and it is reported as the obtained value X2. In all experiments, leaf area of 10 leaves per section was obtained from non-destructive photos at the beginning and the end of the water treatment analyzed using ImageJ software.

### Transpiration Efficiency and the Threshold for a Decline in Transpiration

A third experiment was conducted under greenhouse conditions at the IBt-UNAM (Cuernavaca, Morelos, Mexico) to determine differences in transpiration efficiency and variations in plant responses to drying soil, i.e., the fraction of transpirable soil water (FTSW) threshold for a decline in transpiration. The transpiration efficiency (TE) experiment was conducted as described previously (Devi et al., 2009; Belko et al., 2012). The mean minimum and maximum temperatures in the greenhouse during the experiment were 20°C and 26°C, respectively. The midday vapor pressure deficit in the greenhouse on nearly all days was 13.4 kPa. Eighteen pots per genotype were sown with a single plant per pot. All pots were maintained under well-watered conditions until the start of the TE measurements at flowering stage. From the 18 pots of each genotype, eight pots were harvested on this date to estimate initial plant dry biomass using the method described above. The remaining 10 pots of each genotype were saturated and allowed to drain. The pots were then placed in plastic bags, and the bags were sealed at the base of the plant stem to prevent soil evaporation. Immediately after bagging, the initial weights of the pots were recorded. All pots were weighed every day beginning at 10:00 am. The plants were divided into two treatments: five for well-watered (WW) and five plants for drought treatment (DS). The pots in the well-watered treatment were maintained as such by daily re-watering up to 100% field capacity. Water-deficit was induced by partially compensating plant water loss from transpiration, i.e., plants were allowed to lose no more than 70 g each day. Therefore, any transpiration above 70 g was added back to the pots, as previously described by Vadez and Sinclair (2001), to allow a progressive development of water-deficit stress over approximately two weeks.

#### Transpiration Efficiency

Plants were harvested after 2 weeks, when transpiration in plants under DS was approximately 10% as compared to WW plants, to determine the final plant dry biomass. Transpiration efficiency (g kg^−1^) was calculated as the difference in total plant biomass between the final and initial harvest, divided by the total amount of water transpired during the experimental period (Devi et al., 2009).

#### FTSW Threshold for a Decline in Transpiration

To determine the fraction of transpirable soil water (FTSW) threshold for a decline in transpiration, the daily transpiration values were normalized as previously described (Devi et al., 2009; Kholová et al., 2010; Belko et al., 2012). First, the daily transpiration rate (TR) for each plant was calculated as the ratio of the transpiration rate of each individual DS plant divided by the average transpiration rate for the five WW plants of that genotype. Secondly, the TR data were normalized by dividing each TR value by the average of the TR value for the first 3 days of the experiment, when there was still no water limitation. This normalization eludes the interference of the differences in size among WS plants, resulting in the normalized transpiration ratio (NTR). The daily FTSW (i.e., the amount of soil water available for transpiration) was calculated every day in each experiment. First, the total transpirable soil water (TTSW) in each pot was calculated as the difference between the initial and final pot weight (Sinclair and Ludlow, 1986); and then, the FTSW values were calculated as FTSW = (daily pot weight − final pot weight)/TTSW. Changes in NTR during the soil drying cycle were expressed as a function of FTSW, which was used as an indicator of stress intensity. Plots of NTR vs. FTSW were generated for each genotype, including individual replicated data on each day from all plants. Non-linear regression analysis was done using Prism 9.0. (Graph Pad, Software, Inc., San Diego, CA) to fit the exponential model presented by Muchow and Sinclair (1991). Also using Prism, a plateau regression procedure was employed to estimate a specific FTSW threshold value where NTR begins to decline. The plateau regression attempts to fit two linear segments where one segment is a plateau at *y* = 1 and the second regression is a linear change in *y* with respect to *x*. A key output from this analysis is the FTSW threshold for the two segments and the confidence intervals for this threshold. In this study, the regression analysis was performed on each replicate plant, and a mean was calculated for each genotype.

### Statistical Analysis

The graphs were made using Prism 8 software. The data from the different traits were subjected to variance analysis (ANOVA) using Minitab 19 software, and the differences between the means were compared by Tukey’s test. Values reported with ∗, ∗∗, or ∗∗∗ are statistically significant at probability levels of 5, 1, and 0.1%, respectively. Values reported with NS do not present statistically significant differences.

## Results

### Shoot Biomass Accumulation, Photosynthesis Traits, and Stomatal Density Experiment

#### Shoot Biomass Accumulation

**Figure 1:**
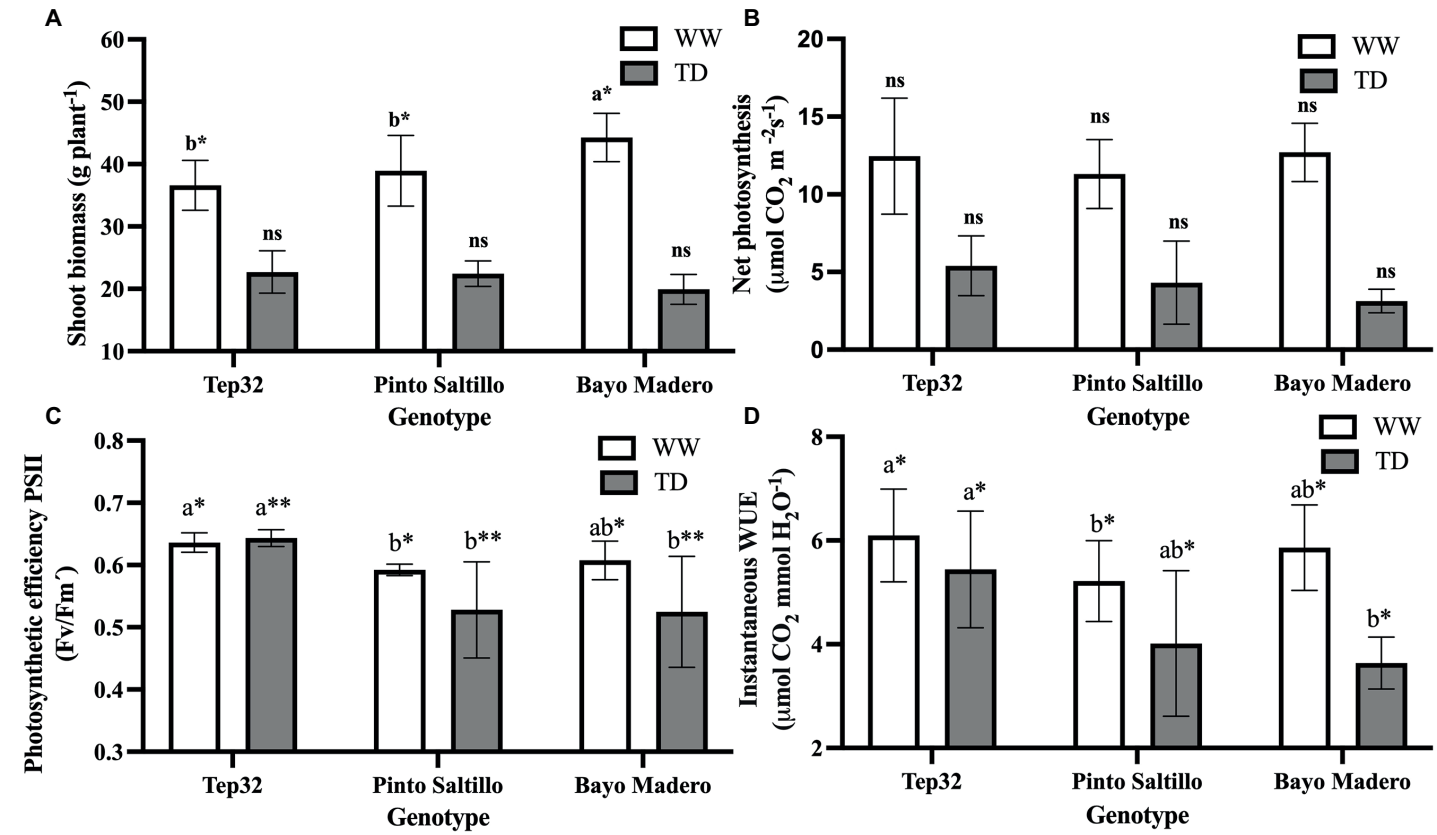
Effect of well-watered (WW) and terminal drought (TD) treatments on **(A)** total shoot biomass, **(B)** net photosynthesis rate, **(C)** photosynthetic efficiency of PSII and **(D)** instantaneous water use efficiency (WUE_i_) of two common bean (PS and BM) and one tepary bean genotypes (“Tep32”). Data are presented as the mean ± standard error of six biological replicates. Asterisks indicate significant differences between genotypes in the same water treatment by *t*-test. ^*^p < 0.05, ^**^p < 0.01, and ns not significant. Letters indicate statistical significance between genotypes determined with Tukey’s test. The statistical analyses between water treatments are not shown.

Terminal drought treatments affected plant growth and development in all three bean genotypes evaluated, showing significant differences (*p* < 0.05) in shoot biomass reduction across the genotypes. Growth of tepary (“Tep32”) was the least affected by water deficit with a shoot biomass reduction of 38%, whereas “Pinto Saltillo” (PS) and “Bayo Madero” (BM) showed a decrease of 42 and 51%, respectively (Figure 1A). Under well-watered conditions, BM presented the highest accumulation of vegetative biomass, followed by PS and “Tep32.” In contrast, although no significant differences in shoot biomass accumulation were detected under water deficit between the three genotypes, BM showed the lowest accumulation compared to “Tep32” and PS (Figure 1A).

#### Net Photosynthetic Rate

Consistent with shoot biomass penalties, terminal drought led to a decline in net photosynthesis rates across the three genotypes (Figure 1B). No significant differences were observed in net photosynthetic rate between the three genotypes under either well-watered or water deficit conditions. However, in terms of absolute values, “Tep32” had higher net photosynthesis than the two common bean genotypes under both treatments (Figure 1B). Under terminal drought, a trend was noticed, where net photosynthetic values were higher for “Tep32,” followed by PS, whereas the lowest values were obtained for BM (Figure 1B).

#### Photosynthetic Efficiency of Photosystem II in Light-Adapted Leaves

Drought stress not only produced a reduction in net photosynthetic rate, but also affected PSII photosynthetic efficiency (Figure 1C). Chlorophyll fluorescence data related to PSII photosynthetic efficiency suggest that terminal drought affected the efficiency of light-to-energy conversion in both common bean genotypes but not in “Tep32,” which did not show differences in this parameter between well-watered and stress conditions (Figure 1C). Significant differences (*p* < 0.05) were observed in the PSII photosynthetic efficiency between the common bean genotypes and tepary bean under both growth conditions. The common bean genotypes (PS and BM) decreased their photosynthetic efficiency from average values of 0.6 under well-watered conditions to values of 0.5 under water deficit (Figure 1C).

#### Instantaneous Water-Use Efficiency

Genotype-specific responses to terminal drought stress were observed in parameters related to water use such as WUE_i_, transpiration efficiency, and FTSW threshold. Terminal drought caused a drop in WUE_i_, as determined from photosynthetic rate/transpiration rate, when compared to well-watered conditions (Figure 1D). Nevertheless, “Tep32” showed the smallest decrease in WUE_i_ as compared to the common bean genotypes (Figure 1D). Significant differences (*p* < 0.05) in WUE_i_ between the three genotypes were observed under both treatments. Under well-watered conditions, “Tep32” had the highest WUE_i_ followed by BM and PS (Figure 1D). Likewise, under water deficit, “Tep32” maintained the highest WUE_i,_ followed by PS and BM (Figure 1D).

### Stomatal Traits and Leaf Size in Response to Water Deficit

**Figure 2:**
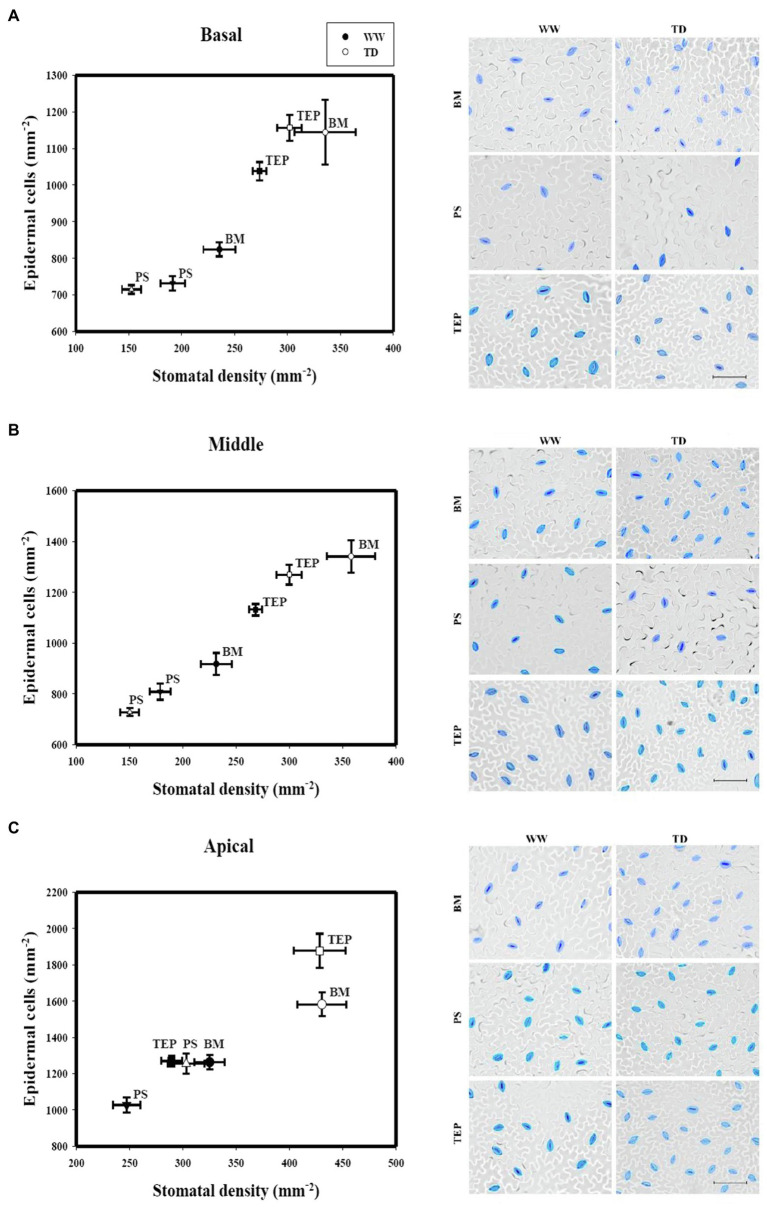
Stomatal density and epidermal cell density of two common bean genotypes (PS and BM) and one tepary bean (“Tep32”) under well-watered (WW) and terminal drought (TD) treatments in three different strata: basal **(A)**, middle **(B)**, and apical **(C)**. Data are presented as the mean ± standard error. Inset: micrographs of representative epidermal impressions with stomata in blue for clarity. Scale bars = 50 μm.

To determine water deficit effects on leaf expansion and stomatal traits, leaf size and epidermal measurements were taken from three strata of the plant canopy: the lower basal section (B), where most leaves have reached maturity; the middle section, containing a mixture of mature and expanding leaves (M); and the apical section, where most leaves are still expanding (A) (Supplementary Figure S3). Leaf size differs between the three genotypes when they are grown under optimal irrigation, with BM possessing the largest leaves and Tep32 having the smallest; however, no significant change was detected in leaf area for the three genotypes in strata B and M between stress and well-watered plants (Supplementary Figure S4). Nevertheless, in B and M strata, under drought, the drought sensitive cultivar BM showed a significant increase in stomatal density (SD; *p* < 0.05 and *p* < 0.001 respectively) and in the number of epidermal cells (*p* < 0.001) when compared to well-watered conditions, whereas leaves of the drought resistant cultivar PS, in which the values for both parameters were significantly lower, showed a significant decrease in both (*p* < 0.05). Tep32 leaves in these strata showed only a significant increase in the number of epidermal cells (*p* < 0.01; Figures 2A,B).

Under the same stress conditions, the top stratum (A) leaves of BM, PS, and Tep32 increased their SD (*p* < 0.001, *p* < 0.05, and *p* < 0.001, respectively) and number of epidermal cells (*p* < 0.001, *p* < 0.01, and *p* < 0.001, respectively; Figure 2C), with the highest difference between well-watered and drought conditions for BM and Tep32, showing again a high correlation between the changes in each of these two parameters, and confirming that leaf SD responses are affected by the leaf growth stage. These data also suggest that for common bean cultivars drought resistance correlates with a lower SD, whereas tepary bean SD may not follow the same pattern.

**Figure 3:**
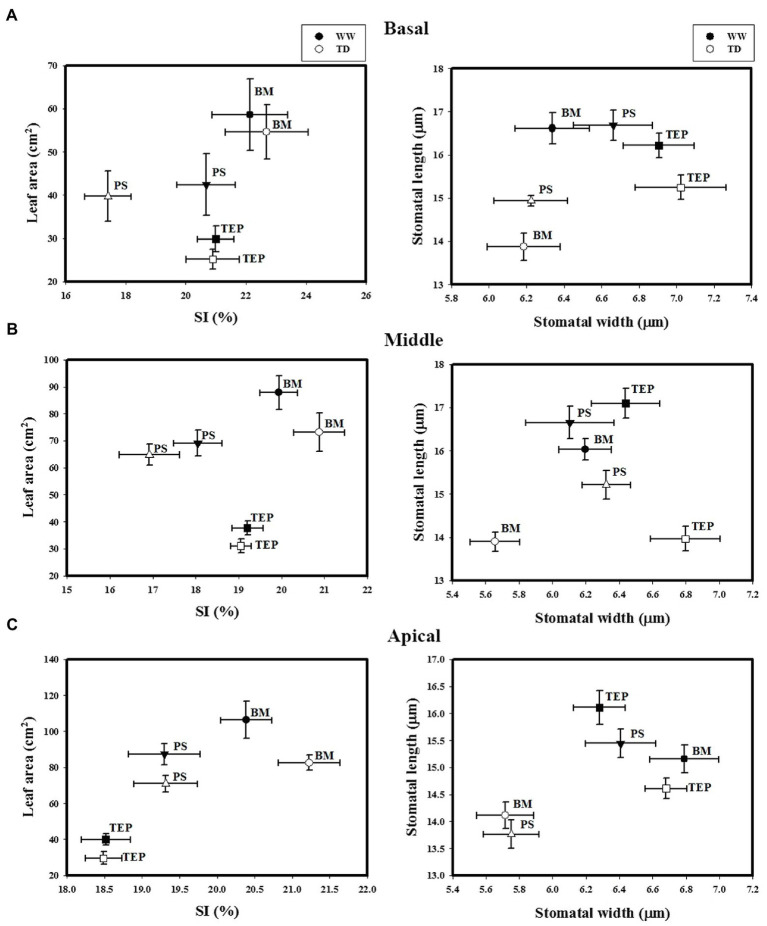
Stomatal index (SI) and leaf area (left) and stomata width and stomata length (right) of two common bean genotypes (PS and BM) and one tepary bean (“Tep32”) under well-watered (WW) and terminal drought (TD) conditions in three different strata: basal **(A)**, the middle **(B)**, and apical **(C)**. Data are presented as the mean ± standard error.

Stomatal index (SI) in B, M, and A strata leaves did not change in either BM or Tep32 under water deficit as compared to well-watered conditions, while Pinto Saltillo’s SI decreased significantly in the B stratum (*p* < 0.05; Figure 3A). BM had the highest SI values and PS had the lowest (Figure 3). In the A stratum, a different SI pattern was obtained between genotypes (Figure 3C). Even though BM leaves maintained higher SI values under both conditions, there was no significant change observed in SI or in leaf area at the apex. Whilst Tep32 and PS SI values also remained similar in the two growth conditions, they both had significantly reduced leaf areas (Supplementary Table S1). These comparative analyses indicate that high SI is associated with BM’s drought sensitivity, and not necessarily to the leaf area of these genotypes.

Stomatal size (width and length) was evaluated in all strata leaves of the three genotypes. For all three genotypes, stomatal length reduced in response to water deficit across all strata; the greatest reduction for BM and PS in stomata of mature leaves (B), for Tep32 and BM in the M stratum, and for PS and Tep32 in the section with younger leaves (A) (Figure 3). However, stomatal width did not change in the basal section (B) between the conditions tested, while in the M stratum a decrease in this parameter was detected only in BM stomata under drought. In the A stratum, BM and PS stomata showed a width reduction in a similar range, whereas for Tep32 no significant change was observed (Figure 3). In general, the analysis of these parameters show that water deficit promotes a reduction in stomatal size in all genotypes, thereby preventing water loss.

### Transpiration Efficiency and the Threshold for a Decline in Transpiration

#### Transpiration Efficiency

**Figure 4:**
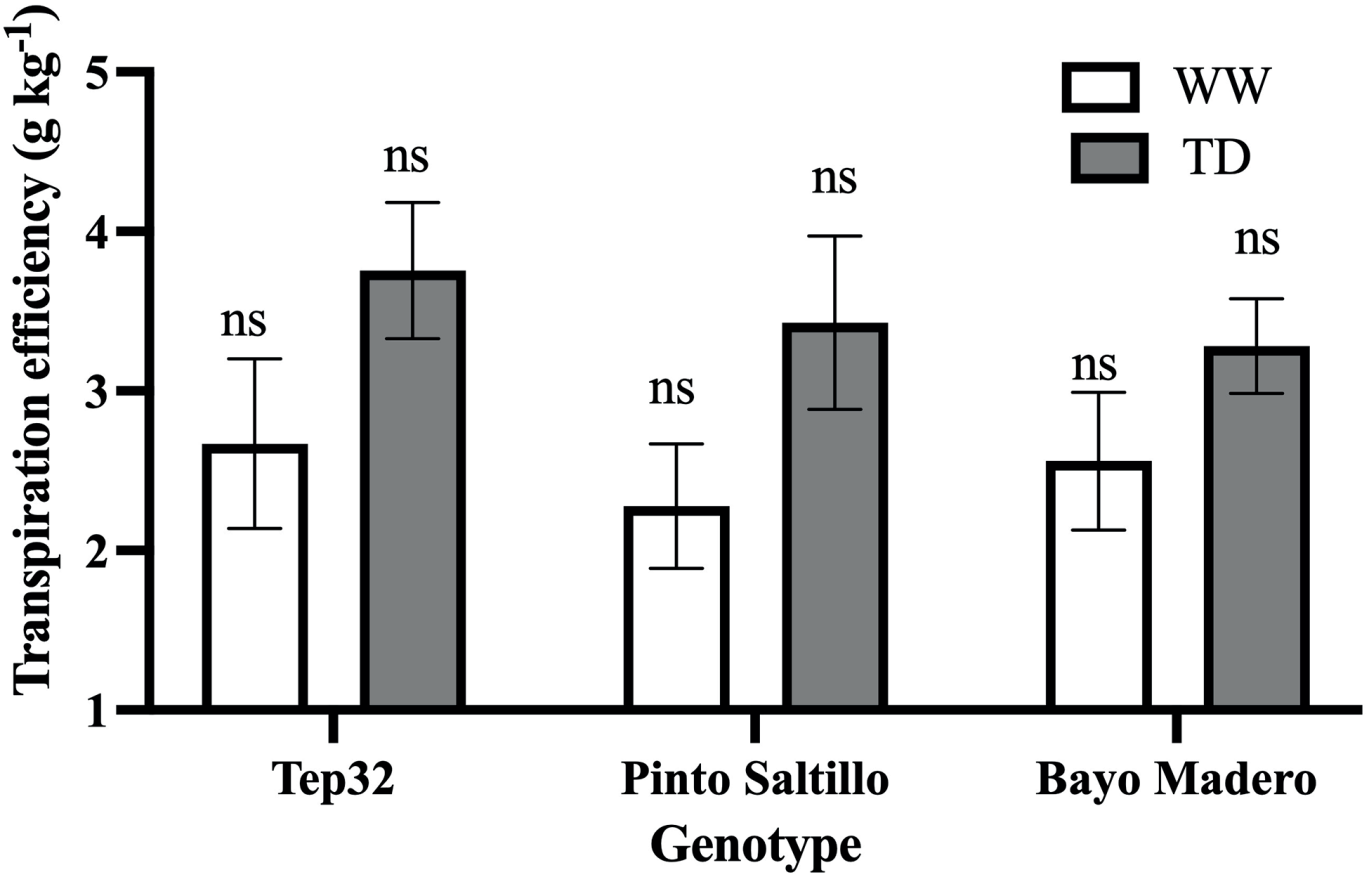
Transpiration efficiency of two common bean (PS and BM) and one tepary bean (“Tep32”) genotypes, under well-watered (WW) and terminal drought (TD) treatments. Data are presented as the mean ± standard error of six biological replicates. “ns” corresponds to not significant (*p* < 0.05).

As shown in Figure 4, all three genotypes responded to drought by increasing their transpiration efficiency (TE), calculated as the change in above-ground (shoot) biomass by transpired water over 15 days. BM, the drought sensitive cultivar, increased TE only by 29%, whereas for “Tep32” and PS, the drought resistant genotypes, TE increased by 41 and 44%, respectively (Figure 4). No significant differences were found among the three genotypes under well-irrigated or drought treatments (Figure 4).

#### FTSW Threshold for a Decline in Transpiration

**Figure 5:**
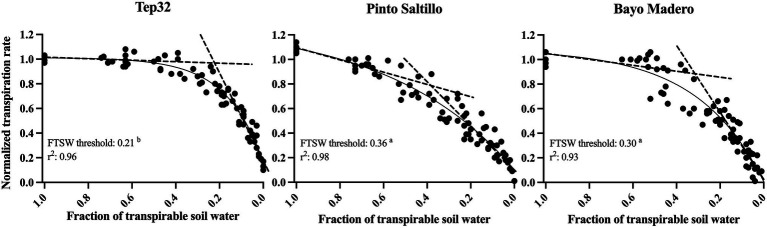
Normalized transpiration rate vs. fraction of transpirable soil water of two common bean genotypes (PS and BM) and one tepary bean (“Tep32”). The solid line in each graph is the regression fit using the inverse exponential model. The dashed lines are the results of the two-segment plateau regression. Letters in the value of FTSW threshold indicate statistical significance between genotypes determined with Tukey’s test.

A consistent pattern in the normalized transpiration rate (NTR) response to soil drying was observed among all replicate plants in each genotype (Figure 5). When the soil moisture is still at field capacity, the NTR value is 1.0 by definition. Hence, as the soil moisture begins to decrease with the expected concomitant decline of the fraction of transpirable soil water (FTSW), the NTR values decrease until reaching values of 0.1 or less at zero FTSW. For this analysis, the relation between NTR and FTSW values were plotted and the data were adjusted to the plateau regression. Although there was a range between genotypes for the values of the coefficients A and B of the plateau regression, the *r*^2^ of the model was higher than 0.90 for all genotypes (Figure 5). Linear regression of the two plateau segments was used to extrapolate the breakpoint as the FTSW threshold, the point where NTR began to decline. Statistically significant FTSW threshold differences were observed between the three genotypes. For Tep32, the NTR started to diminish at the lowest soil water availability, at an FTSW threshold of 0.21 (Figure 5), whereas the PS cultivar, bred for terminal drought-resistance, showed a rapid NTR decline, starting at an FTSW threshold of 0.36 (Figure 5), and BM, considered as susceptible to drought, showed a slower NTR decrease with a start at an FTSW threshold of 0.30.

## Discussion

### Shoot Biomass Accumulation, Photosynthesis Rate, and Instantaneous Water Use Efficiency

This study aimed to perform a more detailed comparative analysis between Durango common bean genotypes with contrasting terminal drought tolerance, and between these and tepary bean. We therefore selected well characterized domesticated varieties of these species. Although this approach may limit a more general application of the results to some extent, it permits a deeper and reliable phenotypic characterization of water use and stomatal dynamics, generating a basis for future research and a framework for exploration of genetic diversity and resistance to terminal drought.

In general, terminal drought stress reduces growth and biomass accumulation in beans and this was observed in all three genotypes evaluated (Figure 1A). Within the common bean (*P. vulgaris*) genotypes, as expected “Pinto Saltillo” (PS), considered resistant to terminal drought, accumulated greater shoot biomass compared to the susceptible genotype “Bayo Madero” (BM). As PS had a milder reduction of shoot biomass under water deficit compared to BM, the resistance of PS to terminal drought may be related to the selection of mechanisms involved in the improvement of biomass accumulation and to those implicated in growth balance under stress (Figure 1A). These observations are in agreement with the more efficient mobilization of photoassimilates from vegetative organs to the formation of the pod and subsequently to the formation of seeds as previously reported (Rosales et al., 2012).

Differences in shoot biomass accumulation were also observed between the two bean species evaluated (*P. vulgaris* vs. *P. acutifolius*), where the tepary genotype showed the least growth inhibition in response to drought stress (Figure 1A). Various reports have demonstrated that tepary beans are more resistant to drought than common beans, as they evolved under desert conditions in northern Mexico and the southern United States (Rao et al., 2013; Polania et al., 2016a, 2017; Buitrago-Bitar et al., 2021). Consistent with these data, our results showed that this species (“Tep32” genotype) was able to accumulate more biomass than the common bean genotypes under the water deficit treatment simulating terminal drought.

A key characteristic for terminal drought resistance seems to be the translocation of photoassimilates from vegetative structures to pod formation and later to seed formation and filling, as demonstrated in greenhouse and field experiments (Cuellar-Ortiz et al., 2008; Rosales et al., 2012; Beebe et al., 2013; Polania et al., 2016a, 2020; Rao et al., 2017). This indicates that the improvement of photoassimilate accumulation and translocation to pods and seeds might be a useful target trait for the development of drought resistant genotypes. To measure photoassimilate translocation is complex and labor-intensive, however, easier and more rapid phenotypic determination of pod harvest index and other parameters have been developed (Assefa et al., 2013; Polania et al., 2016a). In addition molecular analyses of these processes have extended the association of this trait to terminal drought resistance in domesticated common bean (Diaz et al., 2018; Berny Mier y Teran et al., 2019b). The contribution of greater vigor to drought resistance has been reported in different evaluations at the field level (Beebe et al., 2008; Polania et al., 2016a,b; Rao et al., 2017). As indicated by previous studies and by our results, vegetative vigor and reduced inhibition of biomass accumulation may further enhance bean terminal drought resistance. Similar observations have also been reported in heat-tolerant common bean cultivars, where higher biomass allocation to pods has been observed (Omae et al., 2012).

Biomass accumulation is a result of photosynthetic activity (Araus et al., 2002); however, as we show here, the rate of photosynthesis is negatively affected by terminal drought in all three genotypes. Even though the two common beans (PS vs. BM) differ in their seed production under drought, tepary bean showed superior drought resistance even though no significant differences in photosynthetic rate were observed under water deficit between the three genotypes (Figure 1B). This is in agreement with previous studies looking at the impact of drought on bean photosynthesis (Lizana et al., 2006; Rosales et al., 2012; Polania et al., 2020). Nevertheless, it should be noted that under our conditions drought-resistant genotypes exhibited a slightly higher photosynthetic rate compared to the drought-susceptible one (BM), suggesting that these slight differences could contribute to a greater biomass accumulation over time (Figure 1B).

We found limited differences in photosynthetic rate, yet there were significant differences in PSII photosynthetic efficiency between the common bean and tepary species under both well-watered and drought conditions. However, no differences were observed in the photosynthetic efficiency between resistant (PS) and susceptible (BM) common bean genotypes (Figure 1C). Previous studies indicate that tepary beans have high photosynthetic efficiency and minimum damage to their photosynthetic activity under drought (Baath et al., 2020) and heat stress (Traub et al., 2018), but damage is observed under extreme temperatures (Traub et al., 2018). According to our results, this behavior is also observed under the drought treatments we applied. Our gas exchange and chlorophyll fluorescence results strongly support a superior terminal drought resilience in tepary over that of common bean (Rao et al., 2013; Bitocchi et al., 2017; Polania et al., 2017), by showing higher efficiency in light conversion to energy in Photosystem II combined with slightly higher photosynthetic rates.

Water use efficiency (WUE) is a drought resistance trait of great importance across most plant species (Araus et al., 2002; Blum, 2005, 2009; Muñoz-Perea et al., 2007; Easlon et al., 2014; Vadez et al., 2014; Hatfield and Dold, 2019; Leakey et al., 2019). WUE is also a key target trait for crop development in the face of climate change (Hatfield and Dold, 2019). In field trials (Muñoz-Perea et al., 2007; Polania et al., 2016b) and under greenhouse conditions (Rosales et al., 2012) common bean WUE is central to drought resistance. Our results also show that the resistant common bean (PS) genotype has higher WUE than the susceptible one (BM), and that tepary bean (Tep32) WUE is higher than both common bean genotypes (Figure 1D). Tepary’s arid ancestry and desert domestication have arguably provided it with unique mechanisms for enhanced WUE; traits that can be explored in more *P. acutifolius* accessions as well as in related species that have evolved in desert biomes such as *P. parvifolius* (Buitrago-Bitar et al., 2021). Tepary beans have been shown to have superior instantaneous WUE (WUE_i_) even compared to other drought resilient legumes such as mothbean (*Vigna aconitifolia* (Jacq.) Marechal) and guar (*Cyamopsis tetragonoloba* (L.) Taub.; Baath et al., 2020). In addition to a higher WUE_i_, in this study tepary beans were found to be less susceptible to PSII photo-damage under drought, and achieved greater leaf expansion, resulting in greater biomass accumulation (Figures 1A–D), attributed to early vigor and superior WUE under stress (Baath et al., 2020). Field evaluation of carbon isotope discrimination and its relationship with WUE have also determined that tepary bean has lower ^13^C ratios than common bean, indicative of better WUE under drought (Polania et al., 2016a). In summary, our data and previous reports show that tepary bean can maintain higher growth and yields under terminal drought stress through higher photosynthetic efficiency and slightly higher photosynthetic rates combined with higher WUE. This also suggests that a higher RUBISCO efficiency may contribute to a more efficient carboxylation, but this remains to be experimentally validated.

### Transpiration Efficiency and FTSW Threshold for a Decline in Transpiration

Contrasting differences were observed between the two *Phaseolus* species as well as between the two common bean genotypes in transpiration efficiency and stomatal closure threshold (Figures 4, 5). As in instantaneous WUE, tepary beans showed higher transpiration efficiency than common beans under conditions of terminal drought stress (Figure 1D). Tepary bean at the beginning of the soil dry-down period had a higher transpiration rate and sustained it for a longer time than common bean (Supplementary Figure S1), which allowed it greater gas exchange and accumulation of biomass for longer than the common bean genotypes evaluated (Supplementary Figure S2). The two common beans showed contrasting water use strategies over time; the drought sensitive genotype (BM) had higher transpiration rates at the onset of water deficit than both PS and “Tep32,” with a sustained decline after a few days, allowing a greater accumulation of vegetative biomass than PS (Supplementary Figures S1, S2). However, the drought-resistant PS genotype maintained a lower level of transpiration for several days at the beginning of the drought period, with a subsequent decrease, thereby limiting water loss at the cost of biomass gain (Supplementary Figure S2), but with improved yield.

These stomatal closure patterns between and within the bean species are reflected in their FTSW thresholds. The drought resistant common bean PS, responded with earlier yet more gradual stomatal closure (FTSW threshold 0.36), indicating that this cultivar’s earlier water saving allows it to use tap into water later during pod development and filling, critical stages for yield (Figure 5). In contrast, the drought susceptible common bean BM showed an FTSW threshold of 0.30, with high initial transpiration rates and intermediate-to-late stomatal closure (Figure 5), and therefore was unable to save water after the onset of water deficit, rapidly depleting it from the soil. Intriguingly, tepary bean presents an intermediate initial transpiration rate compared to the two common beans and a very different water use strategy over time. With an FTSW threshold of 0.20, tepary maintains transpiration rates for a longer period, declining to transpiration rates similar to those in common beans 13 days after the onset of drought only when the soil is very dry (Figure 5). Altogether, data show that for these two closely related crop species there are three different strategies and patterns of water use.

The strategies for water use shown by the drought resistant genotypes have been observed in other grain legumes and other crop and plant species. The strategy of early stomatal closure and saving soil water showed by the PS cultivar, with high FTSW threshold values, has been observed in drought-resistant chickpea and peanut genotypes (Devi et al., 2009; Zaman-Allah et al., 2011). In arid zone legume crops, such as cowpea, pearl millet (Kholová et al., 2010; Belko et al., 2012), and tepary beans, we observed a strategy with a low FTSW threshold, whereby the plants delay stomatal closure, only reducing transpiration when soil moisture is very low; in this way they maintain gas exchange and carbon fixation for longer and achieve greater biomass accumulation. This drought resilient behavior is also associated with constitutively low stomatal conductance and transpiration rates under well-irrigated conditions, affording them similar gas exchange when water is scarce. This allows the plants to maintain adequate levels of transpiration for longer under water deficit (Kholová et al., 2010; Belko et al., 2012; Vadez et al., 2014), resulting in improved yields. As we see here, tepary bean’s transpiration rates are similar under irrigated conditions and drought for several days (Supplementary Figure S1), showing a decline only when the soil is very dry. Similarly, Tep32 transpiration rates, even in well-watered conditions, are lower than those of the drought susceptible genotype BM, which has the highest transpirational water loss per day under both conditions. This behavior has also been observed among drought resistant and susceptible genotypes of pearl millet and cowpea (Kholová et al., 2010; Belko et al., 2012). The low FTSW threshold shown by tepary bean is arguably combined with many additional traits such as shoot and root architecture which together contribute to the distinctive stress resilience of this species.

### Stomatal Density and Size

Different stomatal responses or strategies have been observed to mitigate the loss of water under drought (Chaves et al., 2016; Pirasteh-Anosheh et al., 2016; Bertolino et al., 2019). In this work, as the studied genotypes present indeterminate shoot growth, we considered the influence of canopy level and leaf growth stage in the stomatal response to water deficit. We evaluated stomatal traits in the leaves of the different genotypes between three different sections, or strata, of the plant canopy: the basal section (B) in which the oldest mature leaves can be found; the middle section (M) in which leaves of different growth stages are located; and the apical section (A), the top of the canopy where leaves are undergoing the most growth and expansion. Overall, the results from this stratified analysis showed that canopy levels and their stages of leaf growth are differentially impacted by water deficit. As plant water status affects cell expansion and consequently leaf size (Welbaum, 2013), genotype-specific responses can be distinguished at the stomatal scale. We observed that in young growing leaves at the top of the canopy there is a significant increase in stomatal density in response to drought in all the genotypes evaluated (Figure 2C). Here, the SD increase correlated with an increase in the number of epidermal cells and no changes in SI (Figure 3C), strongly suggesting that SD increases resulted from the inhibition of cell expansion caused on these leaves by the imposed water shortage.

These data are also consistent with documented observations showing that expanding leaves are more sensitive to water deficit because of their need for a greater amount of water for expansion and for nutrient acquisition (Hsiao and Xu, 2000; Liu et al., 2003; Koch et al., 2019). BM’s greater number of stomata together with its larger leaves likely contribute to the lowest TE and WUE_i_ under drought showed by this genotype (Figures 1D, 2–4). This contrasts with the pattern observed for the drought resistant common bean cultivar PS, which does not show much alteration in the number of epidermal cells in response to drought, suggesting a better growth fine-tuning and/or osmotic adjustment in response to water scarcity. In the two more basal shoot sections, Tep32 showed a small adjustment although this does not seem to be as efficient in the growing young leaves of the topmost section.

A common response to the imposed drought treatments in all three genotypes was a reduction in stomatal length, an effect observed in leaves from all heights in the canopy. As with SD and epidermal cell density, this could be the result of growth adjustments in guard cells and surrounding leaf cells (Kirkham, 2005). The smaller stomata not only reduce stomatal aperture, but could also allow for a more rapid closing-opening response, which may lead to a better balance between the gain of carbon and the loss of water by transpiration (Spence et al., 1986; Lawson and Blatt, 2014; Zhao et al., 2015; Pirasteh-Anosheh et al., 2016). Smaller stomata also promote greater WUE through lower water loss *via* transpiration (Dittberner et al., 2018). For Tep32 in particular, stomatal length adjustment together with the smaller leaf area (Figure 3) may promote higher TE and WUE_i_ under drought (Figures 1D, 4).

Decreased SD is another factor that has been involved in WUE improvement under drought. Various species showing a reduced number of stomata also present lower stomatal conductance, less loss of water and thus an increase in WUE (Wang et al., 2016; Hughes et al., 2017; Bertolino et al., 2019). PS demonstrated significant reductions in SD and SI in the basal and middle canopy leaves in response to water deficit treatments (Supplementary Table S1). This strongly suggests PS maintains phenotypic plasticity even in more mature leaves and is able to directly inhibit stomatal development in response to terminal drought. As with Tep32, this is not observed in the apical leaves of PS, perhaps indicating that the level of stress felt by the youngest leaves is too high to make sufficient adjustments. The mechanisms of control in the basal and middle leaves warrant further investigation.

### Relationship Between Water Use Strategy, Stomata Traits, Leaf Photosynthesis, and Shoot Biomass Accumulation

Superior mobilization of photoassimilates from vegetative structures to pod and seed formation has been shown to be critical for bean drought resistance, as well as for resistance to other abiotic stresses (Cuellar-Ortiz et al., 2008; Rosales et al., 2012; Rao et al., 2017; Polania and Rao, 2019; Polania et al., 2020). Therefore, biomass accumulation contributes toward drought resistance strategies by building enough reserves that will be used for seed formation (Polania et al., 2016a), suggesting that improvement of water use efficiency, photosynthesis, and photoassimilate mobilization might lead to a greater drought resistance in grain legumes.

Our results, using two related bean species, the tepary that was domesticated in arid environments and common beans that were selected in sub-humid to semi-arid environments, demonstrate distinct water use and water deficit response strategies. In response to drought stress, the evaluated tepary accession is able to maintain its stomatal density under stress conditions, as well as a greater presence of smaller stomata, allowing greater versatility in its stomatal control response. Tepary bean maintains transpiration rates for longer, closing its stomata when soil water is almost exhausted. This allows this species to attain greater gas exchange for longer periods, which combined with higher photosynthetic rates, higher PSII efficiency, and higher carboxylation efficiency, ensures biomass accumulation under the inflicted drought treatment. This strategy enables tepary beans to achieve early vigor when soil water is still available, and an effective stomatal closure control when the soil is very dry. This is in agreement with previous observations, where it was shown that tepary bean stomatal sensitivity is advantageous over common beans under restricted water environments (Markhart, 1985); however, further characterization studies of tepary trait diversity in response to drought are required; likewise, exploration of the diversity of these mechanisms in related species such as *P. parvifolius* that also evolved in arid environments and that together with *P. acutifolius* are considered promising sources of resistance to drought (Buitrago-Bitar et al., 2021).

Although our study has focused on above-ground processes, tepary’s extended period of gas exchange, photosynthesis, and growth in response to water deficit may also be attributed to below-ground processes: less vigorous root systems (less branched) with finer, deeper roots, and more metaxylem vessels, allow more efficient extraction of a greater quantity of water from the soil and for longer (Lynch, 2013; Strock et al., 2021). Only when the soil becomes very dry, and its roots can no longer offer advantages, does tepary then drastically close its stomata and reduce transpiration.

The drought-resistant genotype of *P. vulgaris* (PS) at the stomatal level presents a strategy to reduce its stomatal density and index in leaves of the middle and basal strata of the plant, allowing it to reduce transpiration and save water. The early stomatal closure water-saving strategy of the drought resistant common bean genotype at the beginning of the soil drying period halts transpiration, gas exchange, and growth, thereby saving soil water for later use. This strategy, combined with a superior mobilization of photoassimilates to pod and seed formation, confers resistance to drought compared to its counterpart, the drought-sensitive common bean genotype BM. Common bean’s shallow branched roots, although poorly adapted to terminal drought (Beebe et al., 2014; Strock et al., 2021) can acclimate well to conditions of low fertility, more efficiently extracting non-mobile elements such as phosphorus (Beebe et al., 2008; Rao et al., 2016). The shallow roots and low conductance of common bean roots can however be useful for limiting water absorption and thus maintaining limited reserves of soil water and reducing desiccation of root tips and the rhizosphere for sustained water capture later in the season (Strock et al., 2021). This observation strongly supports our results, in which we observe that the water-saving strategy of PS allows better biomass accumulation and yields compared to BM. Whether PS achieves this through combining superficial and well-branched roots along with its stomatal control remains to be evaluated.

It is evident that a broader phenotypic characterization in aspects related to water use, stomatal dynamics, roots, and translocation of photoassimilates, is necessary across the diversity of *P. vulgaris*, *P. acutifolius* and related species. Results of this study show behaviors in a single accession of each species, but future evaluations are required to demonstrate whether they are generalized characteristics in drought resistant genotypes of each species or only some genotypes. For example, regarding the participation of roots, Lynch (2013) and Strock et al. (2021) show that *P. acutifolius* root traits may have evolved in response to arid environments as strategies to favor deeper and non-superficial roots with greater hydraulic conductivity, due to the limited rain and surface water availability. This root phenotype has also been evaluated in *P. vulgaris*, where it has been observed that wild accessions that evolved in arid parts of Mexico show adjustments towards a less vigorous but deeper root system, such as that described in tepary (Berny Mier y Teran et al., 2019a); additionally, wild populations of both tepary and common bean that evolved in arid environments present a reservoir of phenotypic characteristics and genes of interest for drought resistance (Cortés et al., 2013; Buitrago-Bitar et al., 2021). Overall, this demonstrates the diversity to be explored between and within the two species, as well as related species of interest for drought such as *P. parvifolius*. Results of these analyses would provide tools for breeding programs, as well as strategies that can best be adjusted in the fight against drought, either by exploring diversity within *P. vulgaris* (domesticated and wild) using *P. acutifolius* as a model or by introgressing outstanding traits of tepary within common beans (interspecific crosses; Beebe et al., 2013; Burbano-Erazo et al., 2021).

## Conclusion

Water use efficiency and water use patterns throughout the drought period are factors that contribute to drought resistance strategies in beans. By deconstructing bean drought responses, we may start to identify the mechanisms underpinning these phenotypic traits. Our results from the phenotypic characterization of water use of drought-resistant genotypes of two different related species, *P. vulgaris* vs. *P. acutifolius*, showed diversity in their responses, corresponding to their respective adaption to waterless regions, providing strategies for genetic breeding according to drought severity and agroecological zones. The drought-resistant common bean (Pinto Saltillo) genotype shows water-saving and stomatal control strategies, which include a decrease in stomatal density, early stomatal closure, as well as a more efficient control of foliar expansion, which allow it to save available water in the soil, and to resist drought stress. While the tepary bean (Tep32) genotype showed a water spender strategy, which includes maintaining its density of stomata and a late stomatal closure, to improve its foliar gas exchange, resulting in a larger accumulation of biomass. Characterization studies of this kind should be carried out to determine if these traits are a generalized behavior of the species or correspond to specific responses of some genotypes selected under drought conditions. Comparison of domesticated and wild common bean, tepary beans, and their relatives could further tease apart these differential mechanisms and provide unique tools for crop improvement under climate change. Further comparative analyses of combined heat and drought stress will also be invaluable in the face of the climate crisis.

## Data Availability Statement

The original contributions presented in the study are included in the article/**Supplementary Material**, further inquiries can be directed to the corresponding author.

## Author Contributions

JP, VS-C, CCCC, and AAC designed the experiments. JP, VS-C, IG-L, AA-M, and CCCC carried out the greenhouse trials and phenotyping. All authors contributed to the article and approved the submitted version.

## Funding

Funding was provided partially by Programa de Apoyo a Proyectos de Investigación e Innovación Tecnológica (PAPIIT) de la Dirección General de Apoyo al Personal Académico (DGAPA-UNAM) IN204020 grant to AAC. JP was supported by Dirección General de Apoyo al Personal Académico (DGAPA) of the Universidad Nacional Autónoma de México (UNAM) postdoctoral fellowship. CCCC was supported by the European Union’s Horizon 2020 research and innovation program under the European Commission Marie Skłodowska-Curie grant agreement No 700867. AAC and CCCC are also grateful to the Newton Prize and the UK Government’s Department for Business, Energy and Industrial Strategy for funding. VS-C is a student in the Biological Sciences Graduate Program-UNAM, and was supported by a CONACyT fellowship.

## Conflict of Interest

The authors declare that the research was conducted in the absence of any commercial or financial relationships that could be construed as a potential conflict of interest.

## Publisher’s Note

All claims expressed in this article are solely those of the authors and do not necessarily represent those of their affiliated organizations, or those of the publisher, the editors and the reviewers. Any product that may be evaluated in this article, or claim that may be made by its manufacturer, is not guaranteed or endorsed by the publisher.
