# Supplementary material for: Contrasting Phaseolus Crop Water Use Patterns and Stomatal Dynamics in Response to Terminal Drought
Source: Front Plant Sci. 2022 May 31;13:894657. doi: 10.3389/fpls.2022.894657 (PMC9194640; doi:10.3389/fpls.2022.894657)
Supplement: Supplementary file 1 [file Data_Sheet_1.docx]

**Supplementary Material**


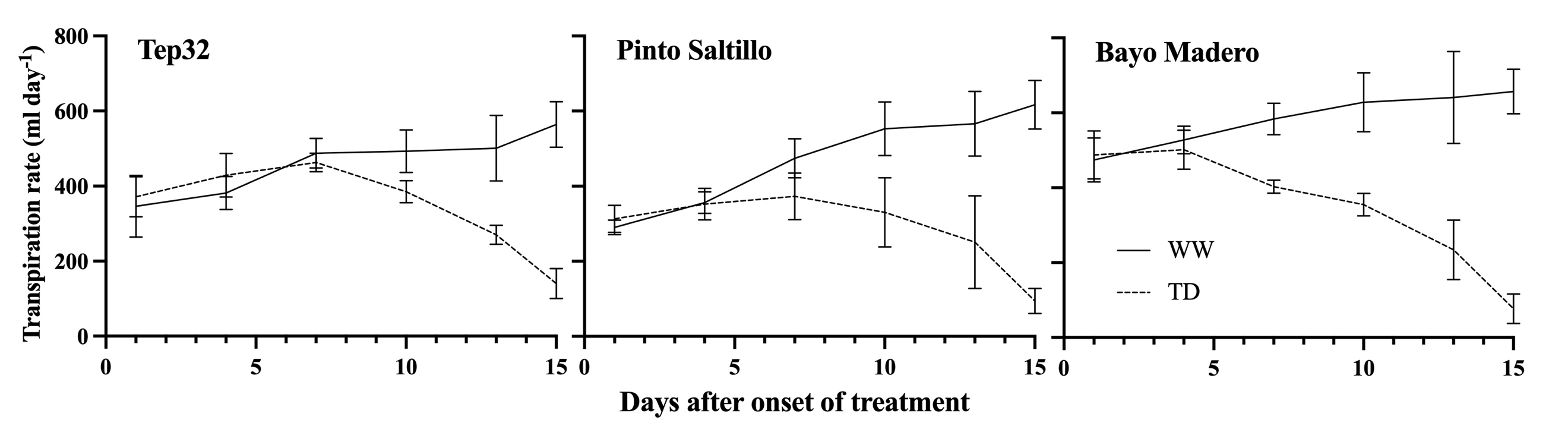


**Supplementary Figure 1.** Daily water transpired by two common bean genotypes (PS and BM) and one tepary bean (‘Tep32’) under well-watered (WW) and terminal drought (TD) conditions. Data are presented as the mean ± standard error of five biological replicates.


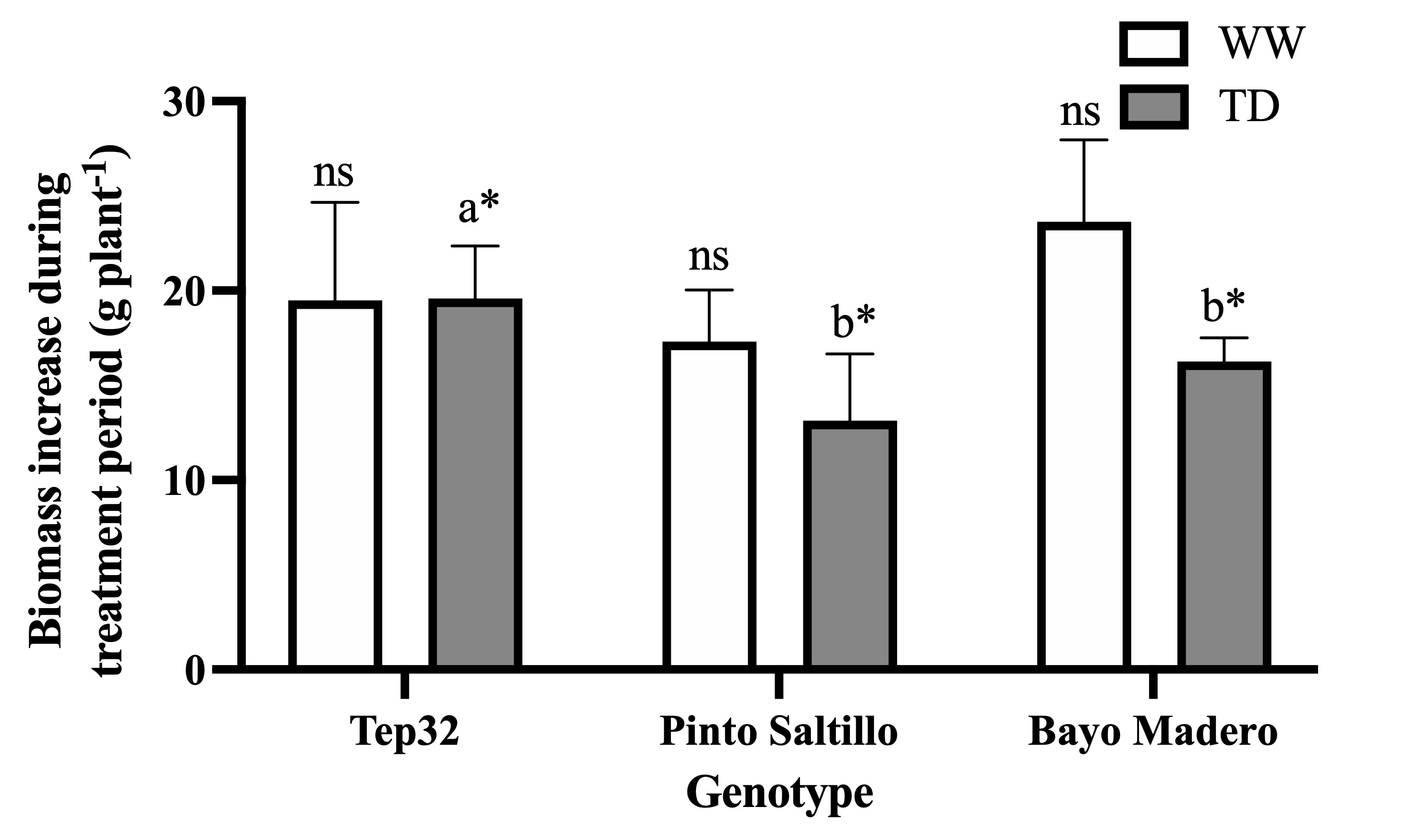


**Supplementary Figure 2.** Biomass of two common bean genotypes (PS and BM) and one tepary bean (‘Tep32’) under well-watered (WW) or terminal drought (TD) conditions. Data are presented as the mean ± standard error of six biological replicates. Asterisks indicate significant differences between genotypes in the same water treatment by t-test. *p < 0.05 and ns not significant. Letters indicate statistical significance between genotypes determined with Tukey's test.


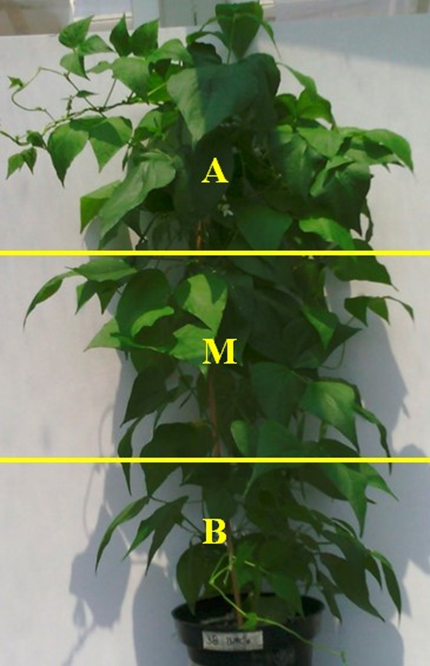


**Supplementary Figure 3.** Picture showing the canopy sections analyzed in this work. Basal section (B), where most leaves have reached maturity; middle section (M), containing a mixture of mature and growing leaves; and apical section (A), where most leaves are still growing.


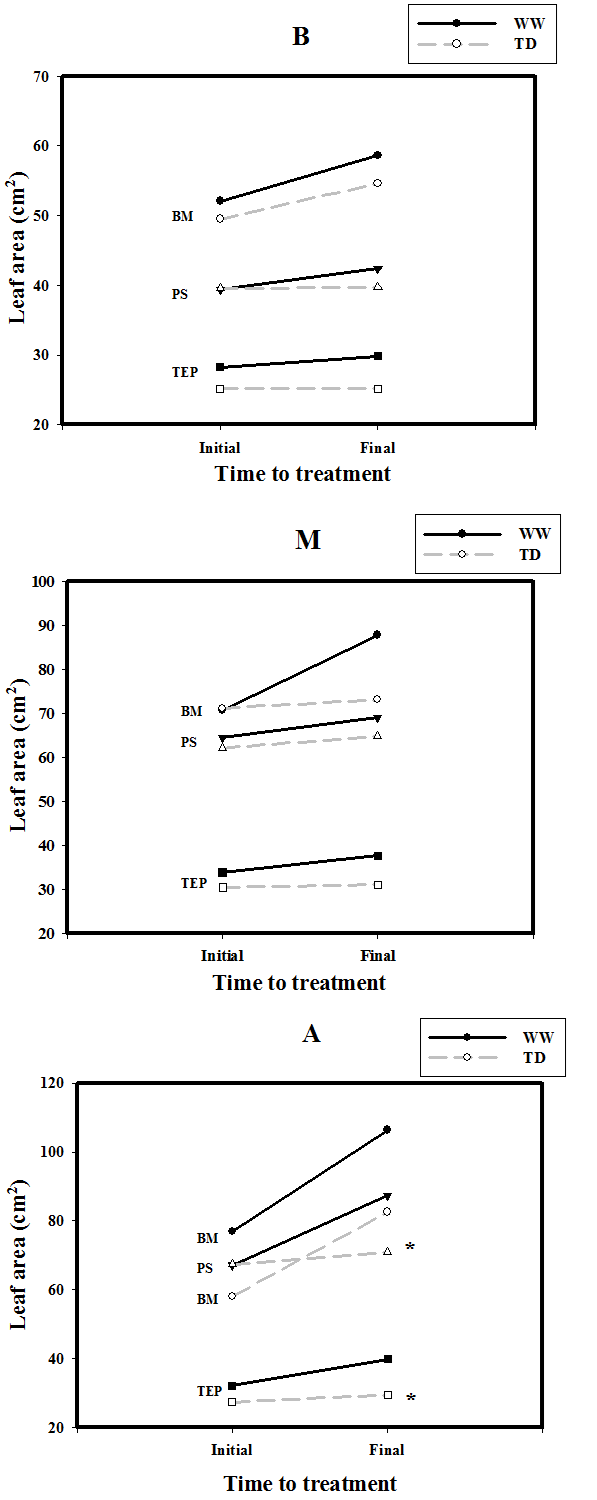


**Supplementary Figure 4.** Leaf area changes in two common bean genotypes (PS and BM) and one Tepary bean (‘Tep32’) grown under well-watered or terminal drought conditions in the three canopy strata: basal (B), middle (M), and apical (A). Data in these graphs show the mean values of 10 leaves per stratum. Asterisks indicate significant differences between treatment by t-test. *p < 0.05.

**Supplementary Table 1.** Stomatal analysis data of two common bean genotypes (PS and BM) and one tepary bean (‘Tep32’) under well-watered (WW) and terminal drought (TD) conditions in three different canopy strata: basal (B), middle (M), and apical (A). Data are presented as the mean ± standard error. Asterisks indicate significant differences between treatment by t-test. *p < 0.05, **p < 0.01, ***p < 0.001

| B | BM- WW | BM- TD | PS- WW | PS- TD | TEP- WW | TEP- TD |
| --- | --- | --- | --- | --- | --- | --- |
| Stomatal density (per mm^2^) | 235.78±15.16 | 335.78±28.95* | 191.57±11.41 | 152.63±8.88* | 273.68±6.47 | 302.1±11.65 |
| Stomatal index (%) | 22.13±1.25 | 22.68±1.37 | 20.67±0.97 | 17.40±0.76* | 20.99±0.60 | 20.89±0.87 |
| Epidermal cells (per mm^2^) | 824.21±19.15 | 1144.21±88.32*** | 731.57±19.25 | 714.73±11.87 | 1037.89±25.19 | 1156.84±35.5** |
| Stomatal length (µm) | 16.61±0.36 | 13.87±0.31*** | 16.68±0.34 | 14.93±0.12*** | 16.21±0.28 | 15.24±028* |
| Stomatal width (µm) | 6.33±0.19 | 6.18±0.19 | 6.66±0.21 | 6.22±0.19 | 6.9±0.18 | 7.02±0.24 |
| Leaf area (cm^2^) | 58.61±8.34 | 54.6±6.25 | 42.42±7.12 | 39.69±5.84 | 29.82±2.96 | 25.16±2.29 |
|  | | | | | | |

| M | BM- WW | BM- TD | PS- WW | PS- TD | TEP- WW | TEP- TD |
| --- | --- | --- | --- | --- | --- | --- |
| Stomatal density (per mm^2^) | 231.57±14.56 | 357.89±22.85*** | 178.94±9.71 | 150.52±8.81* | 268.42±6.27 | 300±11.81 |
| Stomatal index (%) | 19.93±0.44 | 20.87±0.59 | 18.04±0.56 | 16.92±0.69 | 19.2±0.36 | 19.05±0.23 |
| Epidermal cells (per mm^2^) | 917.89±43.5 | 1341.05±64.51*** | 808.42±31.42 | 728.42±16.19* | 1131.57±22.69 | 1269.47±38.73** |
| Stomatal length (µm) | 16.03± 0.25 | 13.89±0.21*** | 16.65±0.37 | 15.21±0.33** | 17.09±0.34 | 13.96±0.28*** |
| Stomatal width (µm) | 6.19±0.15 | 5.65±0.14* | 6.1±0.26 | 6.32±0.14 | 6.43±0.2 | 6.79±0.2 |
| Leaf area (cm^2^) | 87.83±6.26 | 73.13±7.16 | 69.05±4.75 | 64.82±3.85 | 37.66±2.51 | 31.06±2.57 |
|  | | | | | | |

| A | BM- WW | BM- TD | PS- WW | PS- TD | TEP- WW | TEP- TD |
| --- | --- | --- | --- | --- | --- | --- |
| Stomatal density (per mm^2^) | 325.26±14.12 | 430.52±22.83*** | 247.36±13.12 | 303.15±17.1* | 289.47±9.76 | 428.42±24.3*** |
| Stomatal index (%) | 20.38±0.33 | 21.22±0.41 | 19.29±0.47 | 19.31±0.42 | 18.51±0.32 | 18.48±0.24 |
| Epidermal cells (per mm^2^) | 1263.15±39.02 | 1582.1±64.89*** | 1027.36±43.13 | 1254.73±56.18** | 1269.47±29.69 | 1876.84±94.72*** |
| Stomatal length (µm) | 15.15±0.25 | 14.11±0.24** | 15.44±0.26 | 13.76±0.26*** | 16.11±0.31 | 14.61±0.19*** |
| Stomatal width (µm) | 6.78±0.2 | 5.71±0.17*** | 6.4±0.21 | 5.75±0.16* | 6.28±0.15 | 6.67±0.12 |
| Leaf area (cm^2^) | 106.33±10.31 | 82.58±4.14 | 87.34±5.8 | 70.94±4.72* | 39.89±3.14 | 29.53±3.43* |
|  | | | | | | |
